# Editorial: New insights into stress coping and resilience

**DOI:** 10.3389/fnbeh.2023.1253170

**Published:** 2023-07-13

**Authors:** Chong Chen, Yuka Kotozaki, Ryo Okubo, Shin Nakagawa

**Affiliations:** ^1^Division of Neuropsychiatry, Department of Neuroscience, Yamaguchi University Graduate School of Medicine, Ube, Japan; ^2^Department of Hygiene and Preventive Medicine, School of Medicine, Iwate Medical University, Morioka, Japan; ^3^Department of Psychiatry, National Hospital Organization Obihiro Hospital, Obihiro, Japan

**Keywords:** resilience, stress coping, interpretation bias, wellbeing, family resilience, neuroimaging, religious coping, emotion regulation

Whereas the ability to solve problems before they occur is an essential skill, many problems, expected and unexpected, do prevail from time to time. These problems, known as “stressors”, create difficult situations to people concerned, causing tension, worry, and occasionally overwhelming feelings. The latter is called “stress” or more accurately “stress responses”. Adaptive stress responses help initiate effective problem coping mechanisms that remove the stressors or in some cases, adapt to unsolvable stressors. Maladaptive stress responses, including the development of helplessness and hopelessness, in contrast, lead to the disruption of normal homeostasis and increase the risk of stress-related pathology, including a variety of neurological and psychiatric disorders (Chen and Nakagawa, 2020). Importantly, a large proportion of individuals do not necessarily demonstrate maladaptive stress responses and develop stress-related pathology even when they encounter relatively strong stressors, indicating the existence of resilience (Feder et al., 2009; Kalisch et al., 2017). As a key personal asset in face of various stressors including disasters, accidents, crisis, and the ongoing COVID-19 pandemic, advancing our understanding of resilience, therefore, is a critical focus of investigation in psychology, psychiatry, and neuroscience. This Research Topic is a collection of 12 articles in these fields that help us gain novel insights into stress coping and resilience.

Consistent with the observation that positive expectancies are associated with resilience against trauma (Gallagher et al., 2020), Elhamiasl et al. found that negative expectancies or interpretations are associated with anxiety. Using an online task that consisted of 16 ambiguous health-related scenarios that can have both a safe and unsafe interpretation, Elhamiasl et al. found that illness-anxious individuals tend to make more negative interpretations of ambiguous body symptoms. This negative interpretation bias may be a key cognitive mechanism underlying illness anxiety and a potential interventional target for boosting resilience. Surzykiewicz et al. found that religious coping, or turning to religion for coping in stressful situations, may be another strategy for improving wellbeing. Matsuzaki et al. studied resilience in elementary and junior high school students and found that while some resilience factors such as stubbornness are still to be formed, factors including problem-solving, emotional regulation, and leadership common to adults already exist at this young age.

The COVID-19 pandemic represents a typical example of stressor and has been associated with worsened mental health (Chen et al., 2023). Several studies have tried to identify factors contributing to resilience under the pandemic. Maftei et al. reported that during the pandemic, adolescents' social media use as an active coping strategy may help improve wellbeing. Cognitive mechanisms of such benefits include associating social media with positive feelings and expectations of receiving gratification. Employing data from a birth cohort, Dalhof et al. reported that over the course of the COVID-19 pandemic, children showed increased emotional problems, which, however, were more emphasized in those with a mother having experiences of childhood maltreatment.

Whereas parental adverse childhood experiences may cause such vulnerability, parental involvement during childrearing helps cultivate resilience. Cheng et al. found that the when parents of children with autism experience more physical and mental symptoms during the pandemic, their family quality of life worsens. Via parental involvement, such as involvement in school activities, homework, extracurricular activities, hobbies and interests, and monitoring child's life details, however, parents can to some extent restore their family qualify of life. This is consistent with the findings that parent-child interactions, for instance, via conducting physical activities together, help improve family relations during the pandemic (Koga et al., 2023). Furthermore, Cheng et al. found that risk perception of infection and an optimistic attitude toward the pandemic (termed pragmatic hopefulness by the authors) also contribute to better family quality of life, via enhanced parental involvement.

As such, resilience exists not only at individual level, it also exists at family level. Family resilience involves activation of coping strategies at the family level via, for instance, communication and shared decision-making. Family resilience has been considered especially important for cancer patients and their families (Faccio et al., 2019). Within such a context, Almeida et al. evaluated the psychometric properties of the Portuguese version of the Family Resilience questionnaire—Short Form in women with breast cancer.

Four studies attempted to advance our understanding of the brain substrate of resilience with functional magnetic resonance imaging (fMRI). Sugiura et al. found that in response to negative emotional pictures, resilient individuals with traits of adaptive automatic emotion regulation tend to show decreased activation in the sensorimotor cortex as well as multiple cortical regions including the dorsal executive network and anterior cingulate. In contrast, non-resilient individuals tend to show increased activation in regions including the ventrolateral and dorsomedial prefrontal cortices. These findings indicate that automatic adaptive emotion regulation is characterized by automatic disengagement of deliberative processes, which is consistent with another line of evidence showing that contact with the natural environment achieves mood improving effects via relaxing the prefrontal cortex (Yamashita et al., 2021). Employing a thermal environmental stressor, Kawata et al. identified three components of coping behaviors, including motivational decline that reflects emotion-focusing coping, proactive response that indicates problem-focused coping, and active coping that reflects positive appreciation of the stressor. Using fMRI, they further identified neural correlates for two of these three components. Hirano et al. presented death-related words to older adults and found that leadership, one of eight resilience-related traits, was associated with reduced activation in the right inferior parietal lobule in response to such mortality threat. Setroikromo et al. investigated trauma-exposed Dutch police officers and found that resilient officers were characterized by reduced resting-state functional connectivity of the salience network with multiple prefrontal regions. The authors interpreted this pattern of brain activation as reflecting higher capacity for interoceptive awareness and internal-focused thought that helps initiate higher-order coping mechanisms.

The investigation of the biological mechanisms by which various treatments achieve therapeutic effects for neuropsychiatric diseases such as major depressive disorders may also advance our understanding of resilience. In a review paper, Lyu et al. provided an overview of acupuncture treatment for major depressive disorders and suggested that exosomes, extracellular vesicles released from cells for communication with other cells and transmission of molecules, may be a biological mechanism via which acupuncture works.

Taken together, this Research Topic has provided an excellent example of how the field of resilience can be advanced from different perspectives. We hope the novel insights gained by these articles help attract more researchers and accelerate investigation in this field.

## Author contributions

Manuscript draft: CC. Manuscript revision and approval: all authors. All authors contributed to the article and approved the submitted version.
